# Supplementary material for: Risk Factors in HIV-1 Positive Patients on the Intensive Care Unit: A Single Center Experience from a Tertiary Care Hospital
Source: Viruses. 2023 May 13;15(5):1164. doi: 10.3390/v15051164 (PMC10224348; doi:10.3390/v15051164)
Supplement: Supplementary file 1 [file viruses-15-01164-s001.zip › viruses-2388424-supplementary.pdf]

Supplemental Tables and Figures

Figure S 1: Kaplan-Meier curves of overall survival with (A) Hepatitis B and (B) Hepatitis C co-infection.

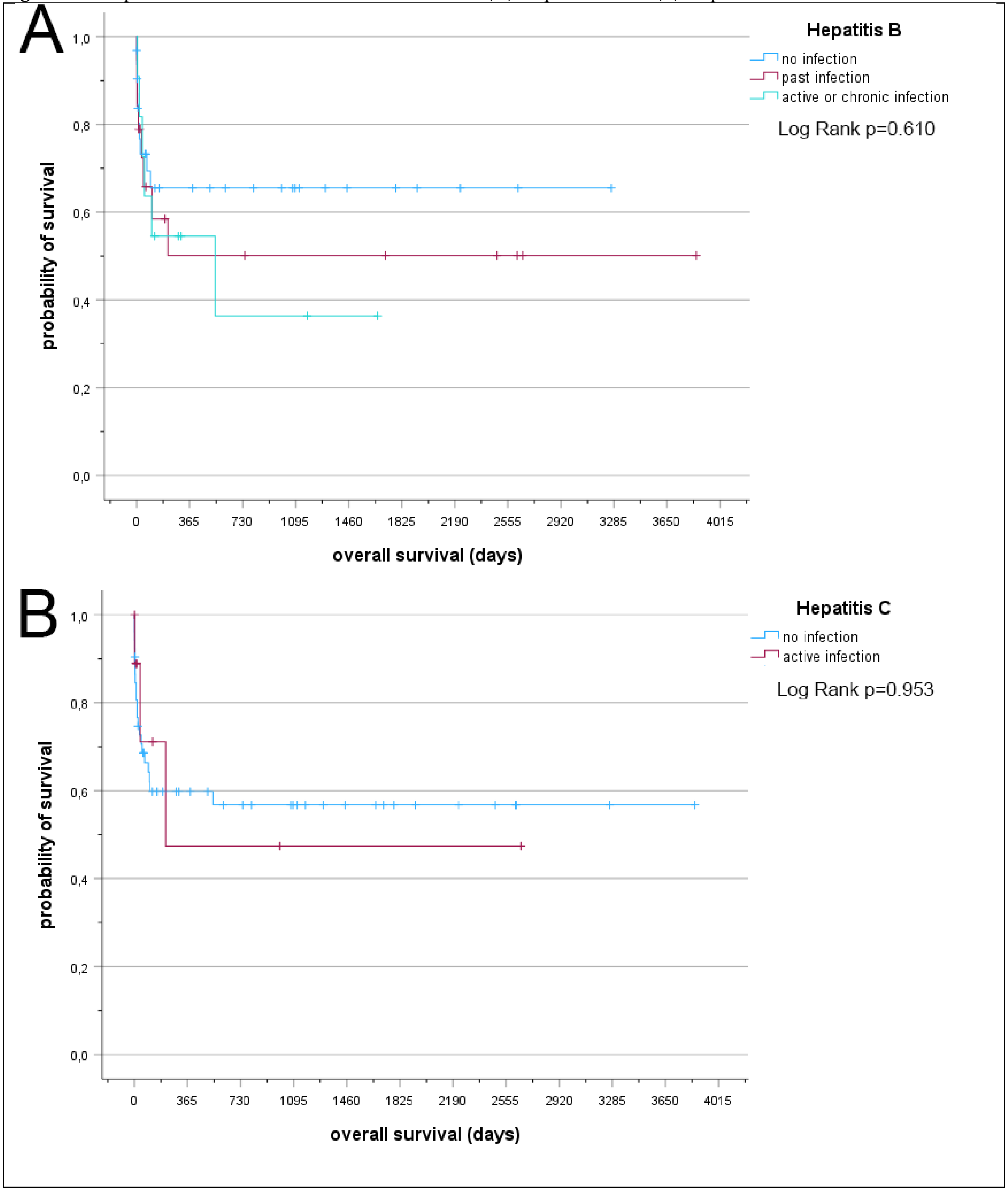

Figure S 2: Area under the curve Receiver operating characteristic analysis for (A) respiratory parameters, (B) laboratory values of blood gas analysis, (C) blood count values, (D) CD4-count, pH level and body mass index, (E) renal and hepatic parameters as well as (F) HIV copy load, co-infections and body temperature.

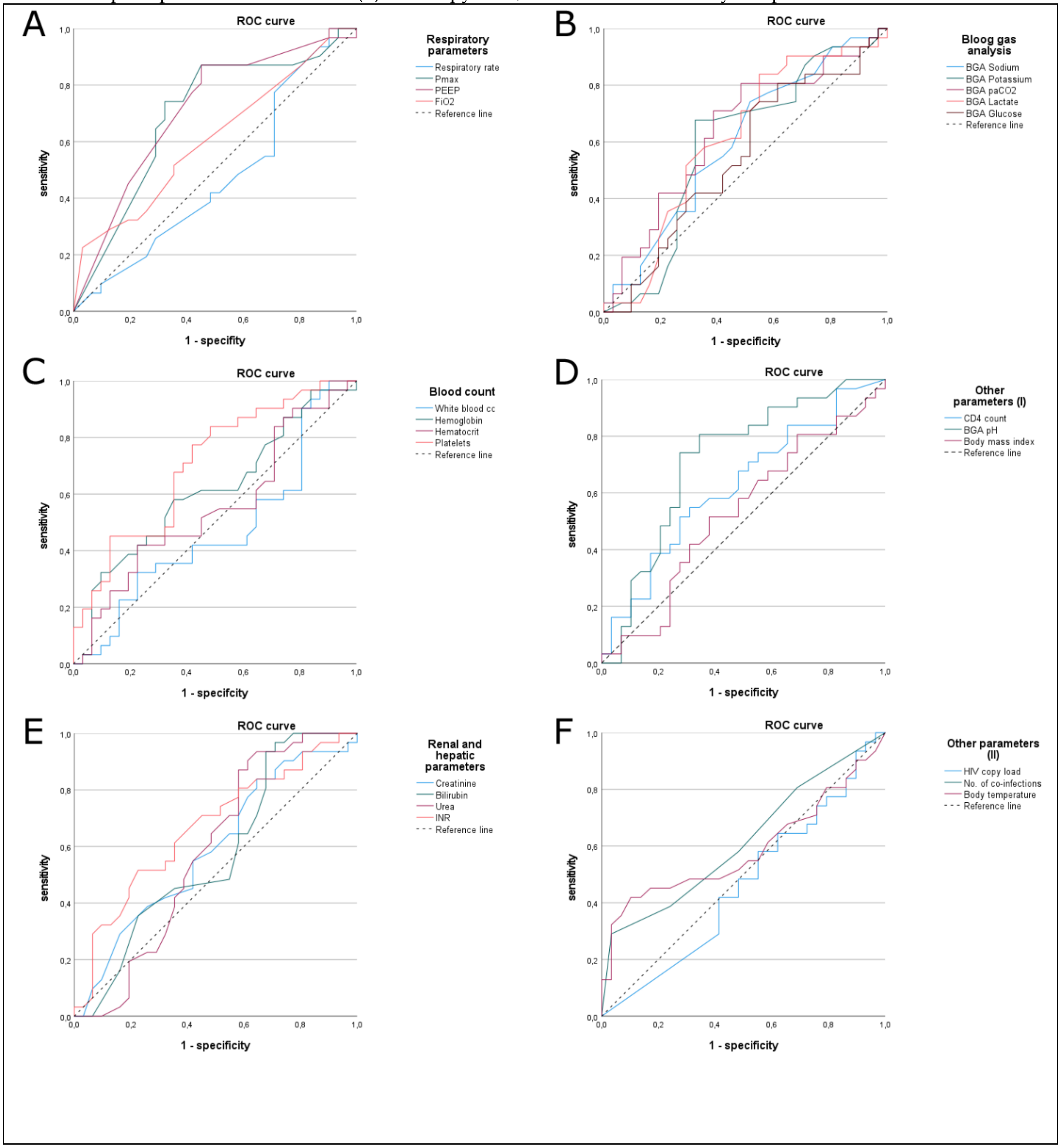

Table S 1: ART regimen in evaluated patients

No	Use of ART	INI	NRTI	PI	NNRTI	EI	Additive
	0 = preexisting<br>1 = newly initiated<br>2 = incompliant use<br>3 = no therapy initiated	0 = no INI<br>1 = raltegravir<br>2 = dolutegravir<br>3 = elvitegravir<br>4 = bictegravir	0 = no NRTI<br>1 = lamivudine<br>2 = emtricitabine<br>3 = stavidune<br>4 = abacavir<br>5 = tenofovir	0 = no PI<br>1 = ritonavir<br>2 = lopinavir<br>3 = darunavir<br>4 = atazanavir<br>5 = fosamprenavir	0 = no NNRTI<br>1 = enfurvitide<br>2 = etravirine<br>3 = efavirenz<br>4 = nevirapine	0 = no EI<br>1 = maraviroc	0 = no additive<br>1 = cobicistat
1	1	1	1	2	0	0	0
2	0	1	0	1,3	0	0	0
3	1	0	2,5	1,3	0	0	0
4	1	0	2,5	1,3	0	0	0
5	1	1	2,5	0	0	0	0
6	1	1	2,5	0	0	0	0
7	1	1	3	0	1	0	0
8	2	0	0	0	0	0	0
9	1	1	0	1,3	2	0	0
10	0	2	1,4	0	0	0	0
11	0	3	2,5	0	0	0	1
12	0	3	2,5	0	0	0	1
13	1	2	2,5	0	0	0	0
14	0	0	1,4	4	0	0	0
15	1	0	2,5	3	0	0	1
16	1	1	2,5	0	0	0	0
17	2	0	0	0	0	0	0
18	0	0	2,5	3	0	0	1
19	0	3	2,5	0	0	0	0
20	0	4	2,5	0	0	0	0
21	2	2	2,5	0	0	0	0
22	1	1	2,5	0	0	0	0
23	0	4	2,5	0	0	0	0
24	1	0	2,5	1,5	0	0	0
25	1	0	2,5	1,2	0	0	0
26	2	0	1,4	4	0	0	0
27	1	0	1,3	2	0	0	0
28	3	0	0	0	0	0	0
29	0	0	1,4	0	3	0	0
30	0	0	1,4	1,4	0	0	0
31	0	1	1	0	3	0	0
32	1	0	1,4	1,3	0	0	0
33	1	0	1,4	1,3	0	0	0
34	2	1	0	1,3	2	0	0
35	3	0	0	0	0	0	0
36	0	1	0	1,2	3	0	0

Table S 1 (cont.): ART regimen in evaluated patients

No	Use of ART	INI	NRTI	PI	NNRTI	EI	Addi-tive
	0 = preexisting<br>1 = newly initiated<br>2 = incompliant use<br>3 = no therapy initiated	0 = no INI<br>1 = raltegravir<br>2 = dolutegravir<br>3 = elvitegravir<br>4 = bictegravir	0 = no NRTI<br>1 = lamivudine<br>2 = emtricitabine<br>3 = stavidune<br>4 = abacavir<br>5 = tenofovir	0 = no PI<br>1 = ritonavir<br>2 = lopinavir<br>3 = darunavir<br>4 = atazanavir<br>5 = fosamprenavir	0 = no NNRTI<br>1 = enfurvitide<br>2 = etravirine<br>3 = efavirenz<br>4 = nevirapine	0 = no EI<br>1 = maraviroc	0 = no additive<br>1 = cobicistat
37	0	1	1	0	3	0	0
38	0	0	2,5	0	4	0	0
39	1	1	0	1,3	0	0	0
40	0	0	2,5	1,3	0	0	0
41	1	0	2,5	1,3	0	0	0
42	0	1	2,5	0	0	0	0
43	0	1	2,5	0	0	0	0
44	1	0	2,5	1,3	2	0	0
45	3	1	2,5	0	0	0	0
46	3	3	2,5	0	0	0	1
47	1	0	2,5	1,3	0	0	0
48	1	0	2,5	1,3	0	0	0
49	0	1	2,5	0	0	0	0
50	1	1	2,5	0	0	0	0
51	1	1	0	1	2	0	0
52	0	0	1,4	0	0	1	0
53	1	0	2,5	1,3	0	0	0
54	0	1	0	0	0	1	0
55	0	1	3	1,2	0	0	0
56	0	1	0	1,3	2	0	0
57	0	1	2,5	0	0	0	0
58	0	0	2,5	3	0	0	0
59	0	1	0	1,3	2	0	0
60	2	0	2,5	0	3	0	0
61	2	3	2,5	0	0	0	1
62	0	4	2,5	0	0	0	0

ART: antiretroviral treatment; INI: integrase inhibitor; NRTI: nucleoside reverse transcriptase inhibitor; PI: protease inhibitor; NNRTI: non-nucleoside reverse transcriptase inhibitor; EI: entry inhibitor; if the incompliant use of a preexisting ART was not sufficiently reproducible, all terms were documented as ‘0’.

Table S 2: Comparison of gender differences and comorbidities and disabilities at ICU-admission

Gender		Female n=14		Male n=48		
		n	in %	n	in %	p
Age (years)	[mean ± SD]	43.7 (± 12.9)		47.1 (± 11.3)		*0.392
BMI (kg/m²)	[mean ± SD]	22.1 (± 7.0)		22.3 (± 4.8)		*0.897
CD4-count (cells/ µl)	[mean ± SD]	125.1 (± 125.7)		176.1 (± 278.6)		*0.342
HIV-copies (mio./ µl)	[mean ± SD]	0.823 (± 1.554)		0.479 (± 1.145)		*0.453
Comorbidities						
Co-infections						#1.000
- Yes		11	78.6	39	81.3	
- No		3	21.4	9	18.8	
Hepatitis B						$0.688
- Active or chronic		3	21.4	8	16.7	
- Past infection		3	21.4	16	33.3	
- No infection		8	57.1	24	50.0	
Hepatitis C						#0.431
- Active infection		1	7.1	9	18.8	
- No infection		13	92.9	39	81.3	
Hematologicial neoplasia						#1.000
- Yes		3	21.4	11	22.9	
- No		11	78.6	37	77.1	
Solid neoplasia						$0.706
- Yes, metastasized		1	7.1	2	4.2	
- Yes, localized		2	14.3	4	8.3	
- No		11	78.6	42	87.5	
Myocardial comorbidity						#0.743
- Yes		4	28.6	12	25.0	
- No		10	71.4	36	75.0	
Rhythmological comorbidity						#0.296
- Yes		5	35.7	10	20.8	
- No		9	64.3	38	79.2	
Pulmonary comorbidity						#0.739
- Yes		3	21.4	15	31.3	
- No		11	78.6	33	68.8	
Vascular comorbidity						#0.475
- Yes		3	21.4	8	16.7	
- No		11	78.6	40	83.3	
Arterial hypertension						#0.028
- Yes		7	50.0	8	16.7	
- No		7	50.0	40	83.3	
Renal comorbidity						#1.000
- Yes		6	42.9	19	39.6	
- No		8	57.1	29	60.4	
Hepatic comorbidity						#0.348
- Yes		7	50.0	16	33.3	
- No		7	50.0	32	66.7	
Neurological comorbidity						#1.000
- Yes		8	57.1	26	54.2	
- No		6	42.9	22	45.8	

p-values estimated by * two-tailed student's t-test, #Fisher's exact test, $ χ^2 test

Table S 3: Student's t-tests between subcohorts with and without malignant diseases.

		No malignancy n=39	Malignancy n=23	
		**Mean (± SD)**	**Mean (± SD)**	**p**
Age	(years)	43.4 (± 10.0)	51.2 (± 13.1)	0.018
CD4 count	(/µl)	219.5 (± 298.5)	68.7 (± 73.4)	0.005
HIV copies	(mio /µl)	0.48 (± 1.01)	0.70 (± 1.59)	0.568
SAPS 2	(pts)	62.4 (± 20.3)	65.5 (± 16.7)	0.523
APACHE II	(pts)	23.3 (± 9.0)	25.0 (± 6.7)	0.373
SOFA	(pts)	8.1 (± 5.0)	9.9 (± 5.3)	0.189

Table S 4: Multivariate Cox regression survival analysis (n=60) including HIV viral load and CD4+ cell count.

**Variables**	**30-day survival**		**p**	**60-day survival**		**p**	**Median overall survival**		**p**
specifications	HR	95% CI		HR	95% CI		HR	95% CI	
pH			0.012			0.018			
< 7.31	5.5	1.5-20.6		3.5	1.2-9.7				
≥ 7.31 (index)									
Platelet count			0.019			0.012			0.012
< 164,000 /µl	6.7	1.4-32.7		5.5	1.5-20.9		3.8	1.3-10.7	
≥ 164,000 /µl (index)									
Solid Neoplasm			0.022			0.003			0.036
No solid Npl. (index)									
Localized Npl.	8.6	1.3-55.7	0.024	8.8	1.8-42.1	0.011	4.2	1.2-15.4	0.028
Metastasized Npl.	4.8	0.9-25.0	0.064	5.9	1.5-23.6	0.012	2.7	0.7-10.2	0.139
APACHE II									0.004
< 25 pts. (index)									
≥ 25 pts.							4.7	1.7-13.5	

p-values calculated via foreward likelihoodratio test, inclusion criteria 0.05, exclusion criteria 0.10; HR: hazard ratio; 95% CI: 95 % confidence interval. Factors evaluated: CD4+ cell count (1) (< 50/µl vs. ≥ 50 /µl), CD4+ cell count (2) (< 200/µl vs. ≥ 200/µl), HIV viral load (< 200 copies/µl vs. ≥ 200 copies/ µl), pH (< 7.31 vs. ≥ 7.31); platelets (< 164,000 /µl vs. ≥ 164,000 /µl); vasopressor use at first 24 hours after ICU admission (yes vs. no); worst ventilation setting at first 24 hours after ICU admission (invasive ventilation vs. non-invasive ventilation plus spontaneous breathing); Pmax (< 16.5 cmH2O vs. ≥ 16.5 cmH2O); PEEP (< 7.5 cmH2O vs. ≥ 7.5 cmH2O); Solid Neoplasm (No neoplasm vs. localized neoplasm vs. metastasized neoplasm); SOFA (< 7 pts. vs. ≥ 7 pts.); SAPS 2 (< 59 pts. vs. ≥ 59 pts.); APACHE II (< 25 pts. vs. ≥ 25 pts.). Index parameters are marked via (index).
